# A Plea for Action Based on Knowledge

**Published:** 1917-10-27

**Authors:** 


### HEALTH AND THE STATE.

### A Plea for Action Based on Knowledge.

To advise an individual how to preserve his health, or even to advise him how to regain it when it has been lost, is a comparatively simple undertaking. The problem is concerned only with a single will, and co-operation, not opposition, may usually be assumed as certain. Very different is the position when the scheme of advice has to be directed to the health of a community. Not merely is the question a larger one. It is indeed larger, but, in addition, it is complicated by a crowd of difficulties and oppositions. Public opinion has to be convinced, alike as to the need of the measures proposed and also as to their efficacy, and the process is proverbially a slow one. Vested interests are apt to be disturbed and they readily take alarm, and usually prove themselves tough customers. The cold and critical eye of the economist is turned upon the new scheme, and may judge it forbidden as unduly expensive. In short, the sanitary reformer finds many lions in his path even before his proposals are accepted, and half-measures and routine may be the measure of his triumph. But there are difficulties also from another side. They come from ill-informed and hurried advocates. The evils are manifest and flagrant, and they must be cured quickly. Such-and-such a proposal is a clear and obvious remedy, and it ought to be applied without delay. To hint that either the evil or the proposed cure has not been fully explored seems to minds of this order an evidence of feebleness or half-heartedness. They are impatient for the end, and of the ready way to reach it they have no doubt. Times like the present bring to these rapid and not always discreet reformers a great opportunity.

### The Chance of the Hurried Reformer.

National shortcomings are laid open to the public gaze, and emotional influences render sensitive the public conscience. That something must be done is a general conviction, and dispatch in the doing would be an added virtue. Hence the hurried reformer has his chance, and his wares are displayed in a favourable market. They make a brave show and promise great things. A more or less widespread sympathy is sure to be engaged, for immediate action has always a commanding quality. A proposal for a more complete and thorough inquiry seems a needless and dilatory suggestion, and the enthusiast triumphs all along the line. Past experiences of a similar order with their records of failure are forgotten, and the quick and certain cure for all our social mischiefs is confidently felt to be at hand. Are there not dangers of this kind in the present hour? The nation is alive to the existence of many evils which prior to the disturbing influence of the war had generally been ignored. It has a strong and ardent desire that these evils shall be overcome. Good people charged with excellent intentions, but by no means always well supplied with knowledge, have remedies to propose. In the nature of things they easily obtain a hearing, and their confidence enlists support from their equally equipped fellows and from those who desire to be in the limelight, shine this where it may. The careful and experienced student of social questions has usually a longer and slower road to offer, and he is not quite so cocksure of a splendid goal. His bid for attention may well prove, therefore, a less attractive one, and his counsels of caution, of thoroughness, of verification of facts, and of adequate preparation are lost in the shouting. The event may justify him, as it has done on more than one occasion in the past, but meanwhile he is out of the running.

### A Thorough Study of Social Problems.

It will hardly be surprising if some such fate as is here roughly sketched should befall a very thorough and well-informed study of the social problems of the hour recently placed before the public by Dr. William A. Brend.* The author has enjoyed both a legal and a medical training, and he is without question a serious and capable student both of the evils of our national life and of the various remedies which are offered for their redress. He has made it his business to survey the whole field of public medicine, and he has brought to his task not only an adequate equipment and unwearied industry, but also a high capacity for analysis and for comprehensive judgment. Moreover, he conducts his argument along the strict lines of reasoning, and marshals his evidence with force and skill. His book will not appeal to the sentimental and popularity-hunting amateur, nor to those who have little taste for cold, hard facts. But no one who really desires to face the actual situation can afford to neglect it, and while it concerns all who are interested in the social problems of the day and of the immediate future, it may be specially commended to members of the medical profession. Into their province as informed and experienced guardians of the public health there rush, in all times of emergency, anxious and willing assistants and leaders. Some of these are fully equal to their ambitions, and their co-operation is cordially welcomed. Others merit a more qualified description, and, while set upon doing good, know little of the actual situations which have to be faced. To these, the short cut and the quick remedy are especially dear, and having reached a ready-made verdict they see no reason to bother about the evidence.

### How to Deal with Complex Issues.

As a matter of fact the issues are not simple, but complex, and not to be unravelled by good intentions and excellent motives. Proposed solutions may wear a very plausible air, but unless they are based upon completeness of knowledge and soundness of judgment the benefits they convey prove but temporary, and their permanent effects

* *Health and the State.* By Wm. A. Brend, B.Sc., M.D. (State Medicine). London: Constable and Co., Ltd. 1917. Pp. 354 + xi. Price 10s. 6d. net.

**HEALTH AND THE STATE**—(*continued*).

are decidedly harmful. Dr. Brend has none of these cheap and superficial methods to advocate. Both his plea and his example make for thoroughness in investigation and for the wise adjustment of means to ends. He may not promise the prompt establishment of a heaven upon earth. But he seeks for the facts of the situation, and these alone are the sure-guides to the relief, so far as this is possible, of our present discontents.

### Why Legislation has Failed.

One of the central propositions which Dr. Brend advances is that a good deal of the legislation which has been adopted for the purpose of promoting the health of the community has failed because it has been based upon insufficient investigation, and he considers this process as likely to repeat itself. This is largely the ground on which he rests his advocacy of the establishment of a Ministry of Public Health. The phrase is an attractive one, but many who advocate it have a very hazy idea of its practical interpretation. Perhaps most generally it conveys the notion that all the medical functions of the various Government Departments are to be detached from their existing associations and to be grouped in a new department under a new Minister. Such is not Dr. Brend's proposal. Indeed he regards the suggestion as impracticable, and certain to cause conflict and confusion. What is needed, he considers, is not the union in a common centre of the existing official medical and health activities, but rather their supervision and co-ordination, and this is one of the tasks he would give to the Minister of Health. But by far the more important function of such a Ministry would be investigation and research. It should do for sickness and disease, as these are distributed among the various classes of the community, what the Registrar-General now does for the facts of mortality. To this end the Ministry would receive the whole of the medical statistical work of the Local Government Board, the Board of Education, the Home Office, and the Insurance Commission, and would apply to these records a severe and rigorous statistical inquiry. There would thus result a body of unchallengeable fact upon which legislative and administrative action could be taken. Further, all the medical work so recorded would be subjected to the examination of an impartial and detached critic, who, unconcerned with administration, would escape the pressure of a natural tendency to put the best possible face on every official proceeding. Whatever may be thought of Dr. Brend's proposal on this question, it is at least a carefully considered one, and it gives to the proposed Ministry of Health a motive which demands recognition. The arguments on one side and the other are fairly presented, and the conclusion, we think, will command attention. If it does nothing else, we may be grateful that it at least gives us a proposal in the place of a phrase.

### Causes of Infant Mortality.

An example of the need for a Minister and staff organised for investigation and research may be found in the case of infant mortality. The subject just now is particularly prominent, and various explanations and solutions are advanced with great confidence. Yet even persons who are in positions which seem to command confidence propound views which are in open and flagrant mutual contradiction. The medical officer of the Local Government Board announces infant mortality to be the index of "social welfare and sanitary administration," while from the corresponding official of the Education Office we learn that external surroundings and conditions are of minor importance, and that "the principal operating influence is the ignorance of the mother." Dr. Mary Scharlieb, on the other hand, endorses the courageous proposition that "probably the most frequent cause . . . is syphilis," and Dr. Jane Walker is reported to become furious at the mere mention of maternal ignorance. It well may be that each of these propositions contains an element of truth, but they cannot each be the whole truth, and there is little difficulty in agreeing that the several authorities emphasise the particular factors with which they are specially concerned. The practical harm which follows is that from each diagnosis issue practical proposals for treatment which are thus advanced as competent to meet the whole of the position. A certain amount of good may be produced this way, but on the whole disappointment ensues, and in the meantime energies which might have been wisely applied had they been directed by knowledge have been spent to little or no purpose.

### Maternal Ignorance Considered.

For the moment maternal ignorance is a welcomed explanation of infant mortality, and out of it have come a Notification of Births Act, schools for mothers, schoolgirl classes for "mothercraft," baby shows, and an army of health visitors. Busy ladies are to be found rushing from centre to centre dispensing knowledge which they have somewhat recently acquired. That all this educational, legislative, and administrative apparatus has done some good in individual cases we should be sorry to doubt, but has it effected the purpose for which it was called into being? Still more, Is the supposition which underlies it all a well-established one? There must surely arise some suspicion on the point when we find that infant mortality among the peasants of Connaught is one-half that among the mothers of Kensington or Westminster and one-third that in Bradford—a town in which the cult of maternal tuition has been developed with almost unlimited enthusiasm. Here, in short, is a social problem of great national importance. Authorities who are supposed to know put forward explanations which are obviously born of incomplete and partial views, and it is no one's business to envisage the whole of the position. As Dr. Brend forcibly points out, the collection and study of the statistics which are essential to a complete comprehension of this and other related questions is an expensive and laborious task, and is little likely to lead to emolument or reward. The private individual, and even the philanthropic com-

HEALTH AND THE STATE—(*continued*).

mittee, really cannot face such an enterprise. It can never be fully undertaken save by an officer having access to all the necessary official records and provided with a staff competent to make these records tell the truth, the whole truth, and nothing but the truth. In the meantime we stumble along in the dark or pursue with temporary zeal a path which cannot lead to the goal.

Test of the National Insurance Act.

Another chapter in which Dr. Brend's critical powers are well displayed is that dealing with the National Insurance Act. He tests the Act by what was declared to be its main object—namely, the improvement of the health of the working part of the community, and, judged by this standard, he concludes that no previous measure has ever failed so signally in its primary purpose. He endorses a proposition which has often been advanced in the columns of The Hospital when he asserts that "had it not been for the voluntary hospitals, medical benefit under the Insurance Act would have been a farce." We are glad to note that while he holds that our hospitals must be linked to other organisations engaged in local medical service, he has no sympathy for a policy which would destroy their voluntary character, and his presentation of the argument against nationalisation or municipalisation is admirable in its force and cogency. Altogether his book is a most attractive one, both in its substance and in its method, and we hope it will command a large circulation. If we have emphasised mainly its critical aspect, it must not be concluded that this is its sole ambition. On the contrary, Dr. Brend, while sternly determined not to be led away by half-knowledge, has on many points thought out his subject to definite conclusions, and he is prepared to advocate and to justify a number of confident proposals where facts warrant this attitude. He has performed an excellent piece of work, and we offer him our cordial congratulations.

